# Weight Goals, Disordered Eating Behaviors, and BMI Trajectories in US Young Adults

**DOI:** 10.1007/s11606-021-06702-y

**Published:** 2021-04-19

**Authors:** Jonathan Chu, Kyle T. Ganson, Eric Vittinghoff, Deborah Mitchison, Phillipa Hay, Jennifer Tabler, Rachel F. Rodgers, Stuart B. Murray, Jason M. Nagata

**Affiliations:** 1 Division of Adolescent and Young Adult Medicine, Department of Pediatrics, University of California, San Francisco, San Francisco, CA USA; 2 Factor-Inwentash Faculty of Social Work, University of Toronto, Toronto, ON Canada; 3 Department of Epidemiology and Biostatistics, University of California, San Francisco, San Francisco, CA USA; 4 Translational Health Research Institute, School of Medicine, Western Sydney University, Penrith, NSW Australia; 5 Department of Psychology, Macquarie University, Macquarie Park, NSW Australia; 6 Camden and Campbell Town Hospitals, Campbelltown, NSW Australia; 7 Department of Criminal Justice and Sociology, University of Wyoming, Laramie, WY USA; 8 APPEAR, Department of Applied Psychology, Northeastern University, Boston, MA USA; 9 Department of Psychiatric Emergency & Acute Care, Lapeyronie Hospital, CHRU, Montpellier, France; 10 Department of Psychiatry and Behavioral Sciences, University of Southern California, Los Angeles, CA USA

**Keywords:** BMI, weight goals, disordered eating behaviors, young adult, weight trajectories

## Abstract

**Background:**

Community sample data indicate that weight control efforts in young adulthood may have associations with greater increases in body mass index (BMI) over time.

**Objective:**

To determine the prospective associations between weight goals and behaviors in young adults and BMI trajectories over 15-year follow-up using a nationally representative sample.

**Design:**

Longitudinal cohort data collected from 2001 to 2018 of the National Longitudinal Study of Adolescent to Adult Health.

**Participants:**

Young adults aged 18–26 years old at baseline stratified by gender and BMI category.

**Main Measures:**

Predictors: weight goals, any weight loss/maintenance behaviors, dieting, exercise, disordered eating behaviors. Outcomes: BMI at 7- and 15-year follow-up.

**Key Results:**

Of the 12,155 young adults in the sample (54% female, 32% non-White), 33.2% reported a goal to lose weight, 15.7% to gain weight, and 14.6% to maintain weight. In unadjusted models, all groups have higher mean BMI at 7- and 15-year follow-up. In mixed effect models, goals to lose weight in men with BMI < 18.5 (5.94 kg/m^2^; 95% CI 2.58, 9.30) and goals to maintain weight in men with BMI ≥ 25 (0.44; 95% CI 0.15, 0.72) were associated with greater BMI increase compared to no weight goal. Engaging in disordered eating behaviors was associated with greater BMI increase in men with BMI < 18.5 (5.91; 2.96, 8.86) and women with 18.5 ≤ BMI < 25 (0.40; 0.16, 0.63). Dieting (− 0.24; − 0.41, − 0.06) and exercise (− 0.31; − 0.45, − 0.17) were associated with lower BMI increase in women with 18.5 ≤ BMI < 25. In women with BMI < 18.5, dieting was associated with greater BMI increase (1.35; 0.33, 2.37).

**Conclusions:**

Weight control efforts may have variable effects on BMI over time by gender and BMI category. These findings underscore the need to counsel patients on the effectiveness of weight control efforts and long-term weight management.

## INTRODUCTION

According to national estimates in the USA, 40% of younger adults aged 20–39 years have a weight status classified as obese with body mass index (BMI, kg/m^2^) ≥ 30.^1^ Higher weight status is associated with many biomedical, psychosocial, and economic consequences, and as such, prevention in young adults has become a public health priority.^2^ In order to prevent obesity and conform to established body ideals, young adults may engage in various weight control behaviors.^3–5^ Studies have shown that individuals with BMI ≥ 25 report a higher prevalence of weight control behaviors.^6–11^ Of concern, disordered eating behaviors (DEBs), such as fasting/skipping meals, vomiting, and taking laxatives or diuretics, have been shown to increase future health risks, including eating disorders, alcohol and tobacco use, and depression, and are unhelpful for weight management.^12–14^

Previous longitudinal studies have shown associations between dieting in adolescence and future weight gain, but results have been inconsistent across studies.^15–19^ Recent longitudinal studies in Minnesota found that adolescents and young adults engaging in DEBs had higher BMI at 5-, 10-, and 15-year follow-up.^6, 20, 21^ Though these findings provide important context for weight control behaviors in adolescents, the external validity of the study was limited due to the lack of nationally representative data. Prior studies using nationally representative data from the National Longitudinal Study of Adolescent to Adult Health (Add Health) have shown associations between DEBs and higher BMI in individuals with BMI ≥ 25 at 7-year follow-up. However, these studies did not investigate the role of weight control behaviors in those with BMI < 25 (i.e., adequate or underweight) and also do not examine efforts to maintain weight after a desired weight has been achieved. Further studies are needed to examine the influence of weight control behaviors on long-term health across BMI categories. Doing so will not only elucidate the cognitive and behavioral factors that predict unhealthy transitions between BMI categories but also help identify interventions for prevention.^14^

While most studies have focused on the associations between weight loss/maintenance efforts and BMI change, there is growing interest in how weight gain efforts may impact future health. Previous studies using nationally representative data have shown that weight gain attempts have high prevalence among adolescent boys.^3^ These findings may reflect societal trends in which the ideal male body has become larger and more muscular, such as those represented in action figures and fitness magazines.^22^ In addition, most weight gain attempts were in adolescent boys who were considered adequate weight, overweight, or obese by BMI measures, suggesting implications for weight gain behaviors leading to overweight/obesity and greater risk for future health complications.^3, 23–25^ Additionally, studies have shown that preadolescent Black girls who are underweight endorse higher rates of weight gain attempts than their White counterparts, which may also reflect desires to comply with cultural ideals.^26, 27^ However, research on goals to gain weight is severely limited, and thus, additional study is needed to better understand and utilize effective obesity intervention and prevention methods.

The present study aims to fill the gap in scientific knowledge on the longitudinal effects of weight goals and weight control behaviors, such as exercise, dieting, and disordered behaviors on BMI trajectories, in young adulthood. The objective of this study was to determine the association in a large national sample of young adults between weight goals, weight control behaviors, and BMI at 7- and 15-year follow-up.

## METHODS

### Sample

We analyzed longitudinal cohort data from the National Longitudinal Study of Adolescent to Adult Health (Add Health), a nationally representative study of adolescents followed into adulthood in the USA. The initial adolescent sample (1994–1995, 11–18 years old, Wave I) used systemic sampling methods and implicit stratification to ensure that the high schools (*n* = 80) and middle schools (*n* = 52) were representative of US schools with respect to country region, size, urbanicity, type, and ethnicity. Thus far, there have been five waves of data collection, coordinated by the Carolina Population Center. For this study, we used restricted-use data from Wave III (defined as baseline for this study), collected from 2001 to 2002 when subjects were 18–24 years old, Wave IV (defined as 7-year follow-up for this study), collected in 2008 when subjects were 24–32 years old, and Wave V (defined as 15-year follow-up for this study), collected from 2016 to 2018 when subjects were 32–42 years old. Additional details regarding the study design may be found elsewhere.^28, 29^

In this study, we included all young adults with weight goal data at baseline and excluded young adults who were lost to follow-up at 7-year follow-up (*n* = 2034) or missing data for the outcomes and covariates listed below (*n* = 133). The University of North Carolina Institutional Review Board approved all Add Health study procedures, and the University of California, San Francisco Institutional Review Board deemed this specific project exempt.

### Procedures

At baseline and 7-year follow-up, an interviewer traveled to the home of or to another suitable location for the research subject. Written informed consent was obtained from the subject. Interviews lasted approximately 90 min. Following the interview, interviewers took physical measurements. At 15-year follow-up, participants were either asked to complete a web-based or paper and pencil questionnaire or given an in-person interview. Following the questionnaire, respondents were asked if they would agree to schedule and participate in a home exam administered by a field examiner to attain physical measurements.

### Measures

#### Baseline Measures

##### Weight Goals

At baseline, participants were asked, “What are you currently doing about your weight?” Individuals who reported “trying to lose weight,” “gain weight or bulk up,” “or stay the same weight” were coded as having a weight loss goal, weight gain goal, or weight maintenance goal, respectively. Those who reported “not trying to do anything” about their weight were coded as having no weight goal.

##### Weight Loss/Maintenance Behaviors

At baseline, individuals who reported a weight loss/maintenance goal were further asked to describe whether or not they engaged in specific behaviors to lose/maintain weight during the past 7 days, which included (1) dieting, (2) exercising, (3) fasting/skipping meals, (4) throwing up, (5) taking weight loss pills, (6) using laxatives, or (7) using diuretics in the past 7 days. An affirmative response to any of (1)–(7) was coded as engaging in weight loss/maintenance behaviors. Individuals who self-reported behaviors (3) to (7) were further coded as engaging in DEBs, and those who self-reported throwing up, laxative use, or diuretic use were coded as engaging in purging behaviors. Our measurement of DEBs mirrors previous studies using Add Health data.^8, 9, 14, 30, 31^ These questions were adapted from validated eating behavior measures, such as in the Adolescent Health Survey. They were similar to Project Eating Among Teens (85% agreement ≥ 1 behavior, *r* = 0.76) except that the time frame was 7 days to be consistent with the 7-day time frame of other validated questions in the Add Health survey on physical activity and nutrition.^32–34^

#### Measured at Both Baseline and 7-Year Follow-up

Body mass index (BMI) was calculated using the standard formula weight (kg) divided by height (m) squared (BMI = weight/height^2^). Weight and height were measured by the interviewer. If not measured by the interviewer, self-reported height or weight was used to calculate BMI. At Waves III, IV, and V, 95.3%, 85.3%, and 43.2% of participants had objective height and weight measurements respectively.

#### Covariates

*Age* and *sex* were based on self-report.

*Household income* was based on self-reported parent’s income at Wave I (1994–1995, ages 11–18) based on the question “how much income did you receive from personal earnings before taxes, that is, wages or salaries, including tips, bonuses, and overtime pay, and income from self-employment?” Gaussian normal regression imputation models were used to impute income for the 1638 parents who either refused to answer the income question or stated they did not know, similar to the method used in previous studies.^35, 36^

*Race/ethnicity* was based on self-report, based on the categories suggested by the Add Health survey design: non-Hispanic White, non-Hispanic Black/African American, Hispanic/Latino, non-Hispanic Asian or Pacific Islander, American Indian/Native American, or other.

### Statistical Analysis

Data analysis was performed in 2020 using STATA 15.1. All baseline measures were calculated with weighted data to reflect the representative proportion in the target US population. We charted unadjusted BMI trajectories by weight goal using mean BMI at each wave. We used mixed effect models to assess prospective associations between weight goals and BMI at 7- and 15-year follow-up, using “no weight goal” as the reference group, adjusting for age, household income, race/ethnicity, and the interaction between wave and weight goal, and accounting for sample weighting. Mixed effect models were also used to evaluate the associations between BMI at 7- and 15-year follow-up as the continuous dependent variable and specific (1) weight loss/maintenance behaviors or (2) weight gain behaviors as the independent variable, adjusting for age, household income, race/ethnicity, and the interaction between wave and behavior and accounting for sample weighting. Given previously reported differences in prevalence and health associations of weight goals and behaviors by sex and BMI, outcomes were stratified by BMI < 18.5, 18.5 ≤ BMI < 25, or BMI ≥ 25 and sex.^3, 8, 9^ In sensitivity analyses, all findings were unchanged when further adjusted for baseline BMI. The Benjamini-Hochberg procedure was used to adjust for a false discovery rate given multiple statistical tests.^37^

## RESULTS

**Fig. 1:**
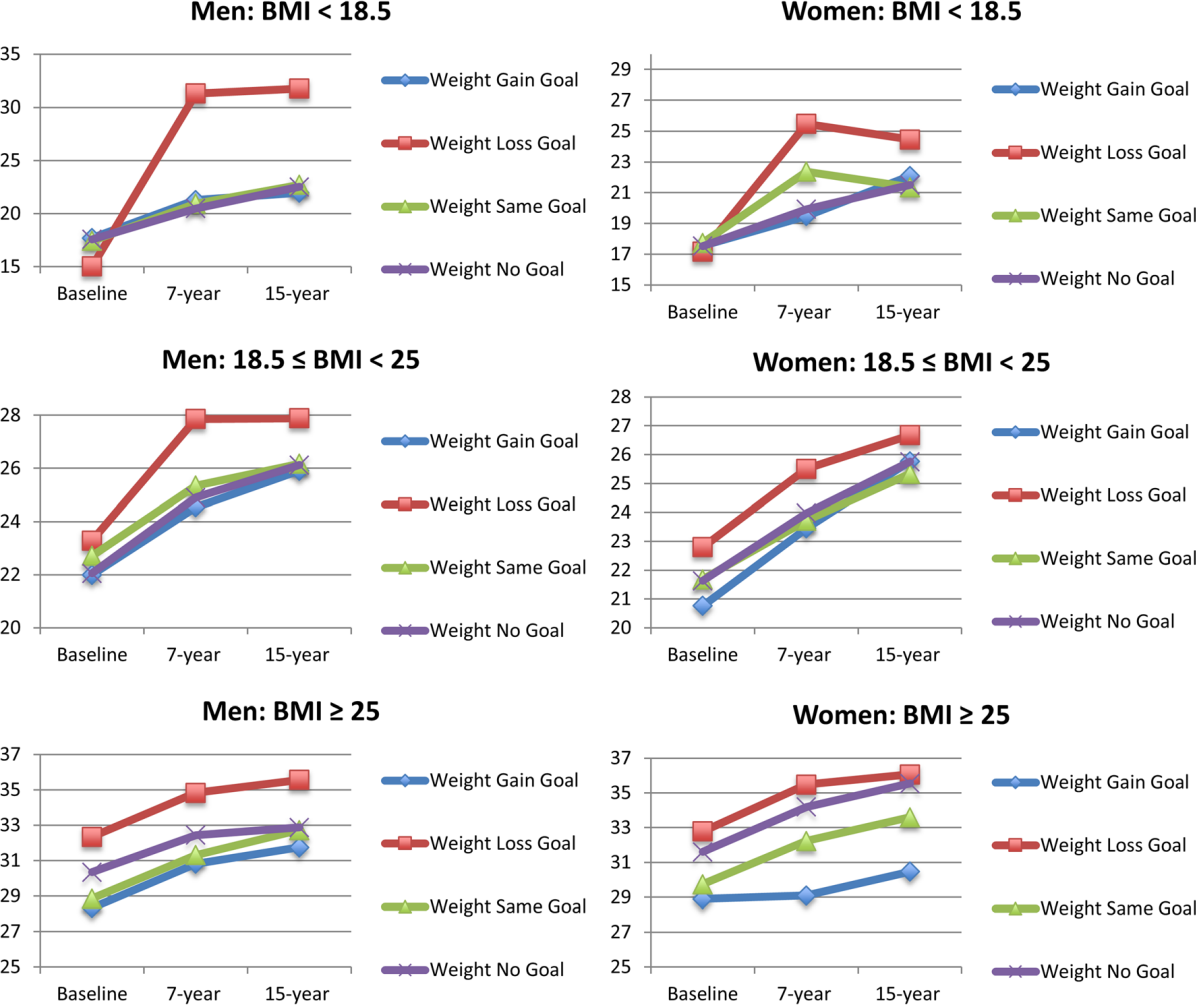
Unadjusted BMI trajectories of men and women over 15 years of follow-up by BMI < 18.5, 18.5 ≤ BMI < 25, and BMI ≥ 25 and by weight goal at baseline.

Baseline demographic and health characteristics of the 12,155 young adults included in the sample are presented in Table 1 by sex and BMI category (< 18.5, 18.5–24.9, or ≥ 25). Overall, 33.2% reported a goal to lose weight, 15.7% reported a goal to gain weight, and 14.6% reported a goal to maintain weight. Figure 1 shows unadjusted BMI trajectories using mean BMI at baseline and at each wave of follow-up. Regardless of sex or weight goal, mean BMI for each group increased over time.

**Table 1:** Baseline Demographic, BMI, Weight Goal, and Behavior Characteristics of Sample from the National Longitudinal Study of Adolescent to Adult Health (Add Health) by Sex

	Total	Male			Female		
		BMI <18.5	18.5 ≤ BMI < 25	BMI ≥ 25	BMI <18.5	18.5 ≤ BMI < 25	BMI ≥ 25
*N*	12,155	118	2,508	2,952	278	3,183	3,116
Demographic characteristics		Mean ± standard error / %			Mean ± standard error / %		
Age (mean ± standard error)	21.80±0.12	21.29±0.31	21.68±0.13	22.13±0.12	21.49±0.18	21.60±0.13	21.83±0.12
Race/ethnicity							
White (non-Hispanic)	68.19%	77.70%	68.00%	67.05%	68.67%	73.75%	63.44%
Black/African American (non-Hispanic)	15.59%	8.71%	15.62%	15.04%	13.50%	11.59%	20.82%
Hispanic/Latino	11.86%	10.67%	11.78%	12.98%	9.49%	9.81%	13.05%
Asian/Pacific Islander (non-Hispanic)	3.10%	2.93%	3.55%	3.10%	6.81%	3.81%	1.57%
American Indian/Native American	0.51%	0.00%	0.36%	0.74%	0.00%	0.27%	0.73%
Other	0.76%	0.00%	0.70%	1.10%	1.53%	0.77%	0.39%
Household income, thousands of US dollars*	45.99±1.37	43.10±2.92	47.01±1.85	43.50±1.40	47.80±3.70	50.85±2.12	42.77±1.29
Body mass index (BMI), kg/m^2^	26.50±0.13	17.61±0.07	22.19±0.05	30.60±0.16	17.55±0.07	21.94±0.04	32.22±0.18
Weight goals							
Lose weight	33.16%	1.40%	5.04%	36.99%	4.11%	29.84%	65.32%
Gain weight	15.71%	58.97%	38.07%	14.42%	32.04%	6.10%	0.49%
Maintain weight	14.62%	2.23%	13.63%	13.07%	14.34%	24.81%	7.54%
Not trying to change weight	36.51%	37.41%	43.26%	35.52%	49.50%	39.25%	26.65%
Weight loss/maintenance behaviors^†^	47.04%	3.63%	17.81%	49.59%	17.95%	53.63%	72.16%
Dieting	19.28%	0.00%	2.94%	19.31%	7.91%	19.70%	37.53%
Exercise	36.46%	2.23%	15.39%	39.38%	14.24%	42.17%	52.64%
Disordered eating behaviors^‡^	11.33%	1.40%	2.66%	10.49%	4.87%	10.50%	23.10%
Fasting/skipping meals	8.91%	1.40%	2.16%	8.54%	4.34%	8.32%	17.63%
Vomiting	0.22%	0.00%	0.07%	0.18%	0.74%	0.31%	0.30%
Weight loss pills	3.41%	0.00%	0.19%	2.59%	0.55%	3.28%	8.20%
Laxatives	0.29%	0.00%	0.29%	0.12%	0.00%	0.47%	0.35%
Diuretics	0.34%	0.00%	0.00%	0.32%	0.00%	0.35%	0.75%

All means and percentages are calculated with weighted data to reflect the representative proportion in the target US population

*Household income was reported in Wave I of Add Health (ages 11–18 years). All other baseline data were taken from Wave III of Add Health (ages 18–26 years)

†Sum of individual weight loss/maintenance may be greater than total due to participants’ ability to select multiple behaviors

‡Disordered eating behaviors were defined as engaging in one of the following weight loss/maintenance behaviors: fasting/skipping meals, vomiting, weight loss pills, laxative use, or diuretic use

### Weight Goals and BMI in Men and Women

Mixed effect models with weight goal as the independent variable and BMI at 7- and 15-year follow-up as the dependent variable, adjusted for age, household income, race/ethnicity, and wave, are presented in Table 2. Among participants with no weight goal at baseline, mean BMI increased by 2.43 kg/m^2^ from baseline to 7-year follow-up and 1.17 kg/m^2^ from 7-year follow-up to 15-year follow-up. Goals to lose weight were associated with greater BMI increases in men with BMI < 18.5, while goals to maintain weight were associated with greater BMI increases in men with BMI ≥ 25. No significant associations were found between any weight goals and BMI change in men with 18.5 ≤ BMI < 25 or in women of all BMI categories.

**Table 2:** Adjusted Change in BMI in US Young Adults with BMI < 18.5, 18.5 ≤ BMI < 25, or BMI ≥ 25 by Weight Goal Compared to Those Without a Weight Goal

Goal	Male	*p*	Female	*p*
	Coefficient* (95% CI)		Coefficient* (95% CI)	
BMI < 18.5				
No goal	REF		REF	
Lose weight	**5.94 (2.58, 9.30)**	**0.001**	0.98 (− 0.20, 2.16)	0.104
Gain weight	− 0.26 (− 0.92, 0.39)	0.427	0.19 (− 0.27, 0.66)	0.414
Maintain weight	0.84 (− 0.53, 2.21)	0.231	0.23 (− 0.40, 0.85)	0.475
18.5 < BMI < 25				
No goal	REF		REF	
Lose weight	0.22 (− 0.10, 0.54)	0.172	− 0.10 (− 0.26, 0.07)	0.261
Gain weight	− 0.07 (− 0.22, 0.08)	0.386	0.25 (− 0.03, 0.53)	0.082
Maintain weight	− 0.16 (− 0.37, 0.04)	0.121	− 0.23 (− 0.40, − 0.06)	0.009
BMI > 25				
No goal	REF		REF	
Lose weight	0.24 (0.02, 0.45)	0.036	0.14 (− 0.10, 0.39)	0.253
Gain weight	0.29 (0.01, 0.58)	0.045	− 0.73 (− 2.10, 0.64)	0.298
Maintain weight	**0.44 (0.15, 0.72)**	**0.003**	0.14 (− 0.30, 0.58)	0.53

Bold indicates statistical significance after Benjamini-Hochberg procedure

*CI*, confidence interval

*Coefficients indicate greater or lower BMI increase per wave of follow-up compared to participants with no weight goal after adjusting for race/ethnicity, age, household income, and wave

### Weight Control Behaviors and BMI in Men

Mixed effect models with weight control behaviors as the independent variable and BMI at 7- and 15-year follow-up as the dependent variable, adjusted for age, household income, race/ethnicity, and wave, are presented in Tables 3 and 4 for men and women, respectively. In young men with BMI < 18.5 at baseline, engaging in disordered eating behaviors, particularly fasting/skipping meals, was associated with greater BMI increase over time. There were no significant associations between weight control behaviors and BMI change in young men with 18.5 ≤ BMI < 25 or BMI ≥ 25.

**Table 3:** Adjusted Change in BMI in US Young Men by Weight Control Behaviors and BMI

	BMI < 18.5		18.5 ≤ BMI < 25		BMI ≥ 25	
	Coefficient* (95% CI)	*p*	Coefficient* (95% CI)	*p*	Coefficient* (95% CI)	*p*
Any weight loss/maintenance behaviors	1.71 (0.45, 2.96)	0.008	− 0.02 (− 0.19, 0.16)	0.858	0.18 (0.00, 0.37)	0.051
Exercise	0.93 (-0.46, 2.32)	0.189	− 0.02 (− 0.20, 0.16)	0.826	0.12 (− 0.06, 0.31)	0.192
Dieting	-	-	0.13 (− 0.25, 0.51)	0.514	− 0.01 (− 0.25, 0.22)	0.908
Disordered eating behaviors^†^	**5.91 (2.96, 8.86)**	**<0.001**	0.23 (− 0.20, 0.65)	0.300	0.21 (− 0.08, 0.50)	0.161
Fasting/skipping meals	**5.91 (2.96, 8.86)**	**<0.001**	0.18 (− 0.27, 0.64)	0.419	0.15 (− 0.17, 0.47)	0.37
Vomiting	**-**	**-**	1.51 (− 1.72, 4.75)	0.359	− 0.92 (− 4.02, 2.19)	0.563
Weight loss pills	**-**	**-**	1.69 (0.40, 2.97)	0.010	0.59 (0.97, 1.11)	0.027
Laxatives	**-**	**-**	− 1.11 (− 3.40, 1.18)	0.343	3.28 (− 0.44, 7.00)	0.084
Diuretics	**-**	**-**	-	-	2.02 (− 0.61, 4.65)	0.133
Purging behaviors^‡^	**-**	**-**	− 0.23 (− 2.10, 1.64)	0.808	0.80 (− 1.07, 2.66)	0.403

Bold indicates statistical significance after Benjamini-Hochberg procedure

Coefficient from mixed effect model. *CI*, confidence interval

*Coefficients indicate greater or lower BMI increase per wave of follow-up compared to participants not engaging in those behaviors after adjusting for race/ethnicity, age, household income, and wave

*†*Disordered eating behaviors were defined as engaging in one of the following weight loss/maintenance behaviors: fasting/skipping meals, vomiting, weight loss pills, laxatives, or diuretics

‡Purging behaviors were defined as engaging in one of the following weight loss/maintenance behaviors: vomiting, laxatives, or diuretics

**Table 4:** Adjusted Change in BMI in US Young Women by Weight Control Behaviors and BMI

	BMI < 18.5		18.5 ≤ BMI < 25		BMI ≥ 25	
	Coefficient* (95% CI)	*p*	Coefficient* (95% CI)	*p*	Coefficient* (95% CI)	*p*
Any weight loss/maintenance behaviors	**0.25 (− 0.31, 0.80)**	**0.008**	**− 0.23 (− 0.36, − 0.09)**	**0.001**	0.12 (**−** 0.12, 0.36)	0.316
Exercise	**−** 0.01 (**−** 0.63, 0.61)	0.978	**− 0.31 (− 0.45, − 0.17)**	**<0.001**	0.12 (**−** 0.10, 0.33)	0.277
Dieting	**1.35 (0.33, 2.37)**	**0.009**	**− 0.24 (− 0.41, − 0.06)**	**0.009**	**−** 0.08 (**−** 0.30, 0.14)	0.473
Disordered eating behaviors^†^	0.03 (**−** 1.12, 1.19)	0.956	**0.40 (0.16, 0.63)**	**0.001**	0.13 (**−** 0.13, 0.38)	0.330
Fasting/skipping meals	0.03 (**−** 1.27, 1.33)	0.960	**0.38 (0.13, 0.64)**	**0.003**	0.25 (**−** 0.04, 0.53)	0.088
Vomiting	**−** 0.53 (**−** 4.00, 2.95)	0.765	0.36 (**−** 1.02, 1.54)	0.694	0.22 (**−** 2.08, 2.53)	0.849
Weight loss pills	0.00 (**−** 2.46, 2.46)	0.999	**0.68 (0.26, 1.10)**	**0.002**	**−** 0.43 (**−** 0.82, **−** 0.04)	0.030
Laxatives	-	-	1.00 (0.05, 1.94)	0.038	**−** 1.20 (**−** 2.80, 0.40)	0.141
Diuretics	-	-	**3.06 (1.82, 4.31)**	**<0.001**	**−** 0.15 (**−** 1.23, 0.94)	0.793
Purging behaviors^‡^	**−** 0.53 (**−** 4.00, 2.95)	0.765	**1.20 (0.50, 1.91)**	**0.001**	**−** 0.29 (**−** 1.16, 0.58)	0.51

Bold indicates statistical significance after Benjamini-Hochberg procedure

Coefficient from mixed effect model. *CI*, confidence interval

*Coefficients indicate greater or lower BMI increase per wave of follow-up compared to participants not engaging in those behaviors after adjusting for race/ethnicity, age, household income, and wave

*†*Disordered eating behaviors were defined as engaging in one of the following weight loss/maintenance behaviors: fasting/skipping meals, vomiting, weight loss pills, laxatives, or diuretics

‡Purging behaviors were defined as engaging in one of the following weight loss/maintenance behaviors: vomiting, laxatives, or diuretics

### Weight Control Behaviors and BMI in Young Women

In young women with BMI < 18.5 at baseline, engaging in dieting to lose/maintain weight was associated with greater BMI increase over time. In young women with 18.5 ≤ BMI < 25 at baseline, dieting and exercise were associated with lower subsequent BMI increase per wave, while engaging in disordered eating behaviors was associated with greater increases in BMI. In particular, of all the disordered eating behaviors examined, engaging in purging behaviors such as diuretic use was associated with the greatest increase in BMI over time. There were no significant associations between weight control behaviors and BMI change in women with BMI ≥ 25.

## DISCUSSION

This nationally representative longitudinal study examined the relationship between weight goals, weight control behaviors, and BMI trajectories over 15 years. We found that, among all young adults, BMI trajectories trend upward in unadjusted analyses regardless of weight goal at baseline, but groups differ by baseline BMI. Goals to lose weight in men with BMI < 18.5 and goals to maintain weight in men with BMI ≥ 25 were associated with greater BMI increase over time relative to other men in those weight categories but with no weight goal. Engaging in disordered eating behaviors was associated with greater BMI increase in men with BMI < 18.5 and women with 18.5 ≤ BMI < 25. Exercise and dieting were associated with less BMI increase in women with 18.5 ≤ BMI < 25, but greater BMI increase in women with BMI < 18.5, relative to women of the same weight categories. Given the prevalence of these weight goals and behaviors in young adulthood, it is imperative to appropriately screen and counsel this population on long-term weight management.

Previous longitudinal studies have provided convergent evidence of the predictive role of DEBs in adolescence with regard to weight gain. The findings in the current study are consistent with results from Minnesota-based studies examining DEBs and BMI at 15-year follow-up and expand upon studies using Add Health examining only 7-year follow-up.^6, 14, 20, 21^ Of note, however, we observe significant relationships regarding DEBs and BMI increase only in young men with BMI < 18.5 and young women with 18.5 ≤ BMI < 25 at baseline. Thus, in our sample, those who engaged in DEBs to lose/maintain weight and experienced greater weight gain were already considered underweight/adequate weight at baseline. These findings in participants with lower BMI may reflect individuals who are weight suppressed, and thus more vulnerable to weight increases over time.^38^ In contrast to the DEBs, endorsement of general exercise and dieting to lose/maintain weight appeared to have the intended effect in young women with 18.5 ≤ BMI < 25. Young women with 18.5 ≤ BMI < 25 who self-reported dieting or exercise to lose/maintain weight gained less weight over time, which deviates from previous studies linking dieting to greater weight gain.^7^ However, dieting may be interpreted in different ways. Individuals engaging in more extreme forms of dieting may underreport the severity of their behaviors in the Add Health survey, which may explain why dieting was found to have associations with greater BMI increase in women with BMI < 18.5. Although the prevalence of these weight control behaviors has been shown to be higher in those with higher BMI, it is interesting to note that the greatest effects on change were on those with lower BMI.^8^ It may be that, in the sample, self-reported behaviors were more extreme in those with lower BMI than in those with higher BMI, thus resulting in different findings. Future studies could examine associations between weight goals, weight control behaviors, and changes in BMI category, such as normal weight to overweight.

We found that, of the disordered eating behaviors, purging behaviors such as vomiting, laxative use, and diuretic use had a greater association with BMI increase than restrictive behaviors such as fasting or skipping meals in women with 18.5 ≤ BMI < 25. While restrictive behaviors were associated with greater BMI increase in men with BMI < 18.5, this may be due to the absence of purging behaviors in that subgroup. Though restrictive eating behaviors were much more common in this sample, these findings may reflect differences in how purging and restrictive behaviors impact normal metabolism and physiology.^39, 40^ Restrictive eating can further promote binge-eating, and these constant fluctuations may disrupt normal metabolism leading to increases in BMI.^39^ There are a number of theories that may explain why purging behaviors may lead to additional BMI gain. Purging behaviors may eliminate excess calories and lead to decreases in metabolism so that, when an individual discontinues purging, they may have increased weight gain.^40^ Alternatively, purging behaviors may be ineffective at weight loss compared to restrictive behaviors, resulting in greater weight gain overall.^41^ Engaging in purging behaviors, often to compensate for binge-eating, may also reflect a more impulsive personality, which has been shown to have associations with higher BMI.^42^ Together, these findings highlight the varying degrees of long-term consequences associated with different disordered eating behaviors. The relationship between weight control efforts and BMI increase is potentially bidirectional, as those who gain weight are also more likely to engage in healthy and unhealthy attempts to lose weight.^11^ Notably, both weight control behaviors have been shown to peak in young adulthood and there is less engagement with them later in adulthood.^3, 9, 31^ Thus, one possible explanation for the greater BMI increase with weight loss goals is the suspension of behaviors in later adulthood as individuals stop trying to lose weight. The greater BMI increase with weight loss/maintenance efforts may also reflect the all-or-nothing mentality of those who engage in extreme dieting, leading to overcompensation after cessation of weight control behaviors.^43^ Additional studies are needed to highlight specific behaviors that may be detrimental to long-term weight control and their implications for long-term health.

The varying findings in men and women warrant discussion of gender differences in weight control efforts. Though weight maintenance goals were associated with greater BMI increase over time in men with BMI ≥ 25, this was not seen in women with BMI ≥ 25. This discrepancy may reflect differences in societal body ideals, as men attempting to maintain their weight may do so to maintain a more muscular physique, while women attempting to maintain their weight may do so to achieve a leaner physique.^3, 4^ Thus, what it means to have a goal of maintaining weight and the corresponding behaviors taken to achieve this goal may differ between men and women. Similarly, dieting and exercise have been shown to have different presentations among men and women, which may contribute to the contrasting findings we present in the current study. For example, previous studies have found that women are more likely to endorse low-fat and low-carb diets and engage in exercises that tone their bodies compared to men.^44^ Furthermore, due to the gendered nature of weight bias, women may feel the need to engage in more extreme behavioral strategies to control their weight at lower BMIs than men, leading to differences in the quantity or quality of dieting and exercise. In general, a greater percentage of women reported goals to lose weight, while a greater percentage of men reported goals to maintain weight in each BMI category. Because the Add Health study design asked those trying to lose weight and maintain weight the same questions regarding weight control behaviors, it may be that the findings in women reflect a greater engagement in weight loss behaviors. Future studies may seek to investigate how engaging in behaviors to lose weight and engaging in behaviors to maintain weight variably shape BMI trajectories.

We acknowledge several limitations in our study. First, the use of self-reported data in asking about weight goals and behaviors may be subject to reporting bias. Furthermore, the use of BMI as a measure should be noted. While BMI has been traditionally used as a surrogate for body adiposity, it does not distinguish between excess fat, muscle, or bone mass.^45^ Self-reported height or weight was used in subjects missing measures; however, the correlation between self-reported height and weight and clinician-administered measures is 0.98–0.99 in general population samples.^46^ Furthermore, this study did not examine the correlation between BMI and more direct indicators of poor physical health. Thus, we cannot state whether those below or above a BMI of 25 were “healthy” or “unhealthy,” respectively. Population data suggests that it is not until a person reaches a BMI of over 35 or 40 that their BMI is associated with significantly poorer health outcomes.^11^ Future studies may consider examining the relationship between disordered eating behaviors and cardiovascular disease risk factors such as hypertension, diabetes, and hyperlipidemia. Lastly, due to the design of Add Health, questions regarding weight loss/maintenance behaviors were restricted to a 7-day time frame, and several of the questions regarding weight goals and behaviors were not asked at all three waves. Thus, we were unable to examine the persistence and severity of weight goals and behaviors over time.

However, the study has several strengths. It used nationally representative longitudinal data of a large (*n* >12,000) sample of young adults with three waves of data collection over 15 years. The study is also among the first to examine the associations between weight goals and BMI change over such a lengthy time period.

In conclusion, over 60% of young adults report a weight goal. Regardless of gender and BMI category, all groups have increased mean BMI over time. Goals to lose weight and goals to maintain weight were associated with greater BMI increase over time in men with BMI < 18.5 and BMI ≥ 25, respectively. Engaging in disordered eating behaviors was also associated with greater BMI increase in men with BMI < 18.5 and women with 18.5 ≤ BMI < 25. These findings highlight the sometimes paradoxical relationships between weight goals and behaviors and BMI, underscoring the need to appropriately screen and counsel individuals on effective long-term weight management.
